# The Standing Committee

**Published:** 1919-04-12

**Authors:** 


### THE STANDING COMMITTEE.

THE following is the list of members of Standing Committee E of the House of Commons to which the Bill has been referred:—

Mr. R. Armitage (Leeds Central), Major the Hon. W. Astor (Parliamentary Secretary, Local Government Board), Major Barnett (St. Pancras, S.W.), Mr. A. R. Barrand (Pudsey and Otley), Mr. F. Briant (Lambeth, N.), Colonel C. R. Burn (Torquay), Mr. T. W. Casey (Attercliffe), Rt. Hon. Evelyn Cecil (Aston), Sir Wm. Watson Cheyne (Scottish Universities), Sir Richard Cooper (Walsall), Captain Colin Coote (Isle of Ely), Mr. Dugald Cowan (Scottish Universities), Captain Dixon (Pottinger), Major John Edwards (Aberavon), Major Glyn (Clackmannan and Eastern), Mr. William Graham (Edinburgh Central), Mr. Joseph Green (Leicester, W.), Captain the Hon. Rupert Guinness (Southend-on-Sea), Mr. R. S. Gwynne (Eastbourne), Sir Charles Hanson (Bodmin), Major Hayward (Seaham), Major Hennessy (Winchester), Rt. Hon. John Hodge (Gorton), Mr. Harry Hope (Stirling and Clackmannan), Mr. Austin Hopkinson (Mossley), Major Howard (Sudbury), Sir Edgar Jones (Merthyr), Major Kelley (Rotherham), Mr. T. A. Lewis (Pontypridd), Major Lloyd-Graeme (Hendon), Mr. C. E. Leo Lyle (Stratford), Mr. L. Lyon (Hastings), Mr. Henry M'Laren, Sir George Croydon Marks (Cornwall, N.), Major-General Sir Newton Moore (Islington, N.), Mr. W. G. Perring (Paddington, N.), General Sir Ivor Philipps (Southampton), Sir Philip Pilditch (Spelthorne), Lieut.-Colonel Nathan Raw (Wavertree), Mr. Robert Richardson (Houghton-le-Spring), Mr. Frederick Roberts (West Bromwich), Sir Samuel Scott (St. Marylebone), Mr. A. Short (Wednesbury), Mr. W. Smith (Wellingborough), Mr. G. A. Spencer (Broxtowe), Sir Charles Sykes (Huddersfield), Mr. John Taylor (Chester-le-Street), Brigadier-General Sir Owen Thomas (Anglesey), Colonel Wedgwood (Newcastle-under-Lyme), Major Wheler (Faversham), Mr. Aneurin Williams (Consett), Mr. Thomas Williams (Swansea, E.), Viscount Wolmer (Aldershot), Sir Robert Woods (Dublin University), Colonel Yate (Melton).

